# Incidence, Prognostic Factors and Survival Outcome in Patients With Primary Hepatic Lymphoma

**DOI:** 10.3389/fonc.2020.00750

**Published:** 2020-05-14

**Authors:** Shi-Long Zhang, Chen Chen, Qian-Wen Rao, Zhe Guo, Xin Wang, Zhi-Ming Wang, Li-Shun Wang

**Affiliations:** ^1^Minhang Hospital, Fudan University, Shanghai, China; ^2^Department of Hematology, Zhongshan Hospital, Fudan University, Shanghai, China; ^3^Department of Internal Medicine, Ophthalmic Hospital of Hebei Province, Xingtai, China; ^4^Department of Acupuncture and Moxibustion, Central Hospital of Shanghai Xuhui District, Shanghai, China; ^5^Department of Medical Oncology, Zhongshan Hospital, Fudan University, Shanghai, China; ^6^Department of Medical Oncology, Xiamen Branch, Zhongshan Hospital, Fudan University, Xiamen, China

**Keywords:** primary hepatic lymphoma, SEER, incidence, treatment, prognosis, nomogram

## Abstract

**Aim:** The objective of our study was to investigate the epidemiologic characteristics, prognostic factors and survival in patients with primary hepatic lymphoma (PHL).

**Methods:** PHL patients diagnosed between 1983 and 2015 were identified from the SEER database. The temporal trend in PHL incidence was assessed using joinpoint regression software. Overall survival(OS) and disease-specific survival (DSS) was evaluated using the Kaplan-Meier method and log-rank test. Univariate and multivariate Cox regression analysis was performed to identify the independent prognostic factors for OS and DSS. Nomograms to predict survival possibilities were constructed based on the identified independent prognostic factors.

**Results:** A total of 1,182 patients were identified with PHL. The mean age was 61.7 ± 17.1 years with a male to female of 1.6:1. Diffuse large B-cell lymphoma (59.8%) was the most common histological subtype. The incidence of PHL steadily increasing by an annual percentage change (APC) of 2.6% (95% CI 2.0–3.2, *P* < 0.05). The 1-, 5-, and 10-year OS rates were 50.85, 39.6, and 30.4%, respectively, and the corresponding DSS rates were 55.3, 47.9, and 43.3%, respectively. Multivariate Cox regression analysis revealed that age, sex, race, marital status, histological subtype, surgery, and chemotherapy were independent prognostic factors for survival. Nomograms specifically for DLBCL were constructed to predict 1-, 5-, and 10-year OS and DSS possibility, respectively. The concordance index (C-index) and calibration plots showed the established nomograms had robust and accurate performance.

**Conclusion:** PHL were rare but the incidence has been steadily increasing over the past four decades. Survival has improved in recent years. Surgery or chemotherapy could provide better OS and DSS. The established nomograms specifically for DLBCL were robust and accurate in predicting 1-, 5-, and 10-year OS and DSS.

## Introduction

Primary hepatic lymphoma (PHL) is a rare malignancy whose pathogenesis is still unclear. It accounts for ~0.1% of hepatic malignant tumors and 0.4% of extranodal lymphoma (1). It was first described in 1998, and Lei et al. (2) defined it as a lymphoproliferative disorder confined to the liver without any involvement of the lymph nodes, spleen, or bone marrow. Most PHL patients present with upper abdominal pain, upper abdominal distention or discomfort. The non-specific clinical presentation includes fever, loss of weight, night sweats, jaundice, and hepatomegaly. Laboratory tests may reveal either a cholestatic or a cytolytic process, with elevated lactate dehydrogenase and alkaline phosphatase in most patients. Imaging tests often reveal an isolated lesion in the liver which is similar to that of liver cancer. The predominant histological type of PHL is non-Hodgkin's B-cell lymphoma, most commonly diffuse large cell type (3). PHL is often misdiagnosed as many other liver diseases, and biopsy is usually performed to make a definite diagnosis (2).

Just as most rare diseases, no unified recommendation has been offered for PHL. Our current knowledge about PHL mainly stems from individual case reports or retrospective analysis with small series. Studies on incidence, treatments, and survival in PHL that conducted on a large population-level have not been reported yet. The Surveillance, Epidemiology, and End Results (SEER) database provides favorable resources for investigating rare malignancies like PHL in the settings where prospective data or clinical trials are limited (4). So far the present retrospective analysis of the SEER database represents the largest and latest PHL cohort in the literature. In this study, we used the SEER database to describe the incidence, prognostic factors, and survival trends of PHL. We also characterize independent prognostic factors associated with PHL and sought to build prognostic nomograms that could assist clinicians to estimate prognosis accurately.

## Materials and Methods

### Patients

Information regarding patients diagnosed with PHL between 1983 and 2015 were extracted from the SEER database via SEER^*^Stat software. The diagnosis of PHL has been controversial (5). To reduce the risk of including secondary liver involvement of terminal systemic or adjacent lymphoma, our study focused on a diagnosis of lymphoma and a primary location confined to the liver with no history of prior tumor diagnosis. International Classification of Diseases for Oncology, 3rd edition (ICD-O-3) histologic codes 9590–9595, 9650–9699, and 9702–9729 were used to identify lymphoma and site specific code C22.0 was to identify lymphoma primarily limited to liver. All eligible patients were selected according to pathologically confirmed diagnosis. Patients were excluded if they had incomplete demographic or clinicopathological, as well as, follow-up information.

The following demographic and clinicopathological variables were included in our analysis: age, sex, race, year of diagnosis, marital status, histologic subtype, surgery, radiation, chemotherapy, survival months, vital status, and cause of death. Race was aggregated into White, Black and others (American Indian/Alaskan Native or Asian/Pacific Islander). To examine the trends in survival of PHL over the past four decades, we also categorized PHL patients based on years of diagnosis, namely 1983–1993, 1994–2004, and 2005–2015. The primary outcomes in our study were overall survival (OS) and disease-specific survival (DSS). OS was calculated as the time from diagnosis to death regardless of any cause, and DSS was calculated as the time from diagnosis to death from PHL.

### Statistical Analyses

The incidence rates of PHL were calculated per 100,000 persons and age-adjusted to the 2,000 US Standard Population using SEER^*^Stat (version 8.3.2). Annual percentage changes (APCs) were calculated using the National Cancer Institute join-point regression analysis program (version 4.5.0.1). Estimated OS and DSS was calculated with Kaplan-Meier method, and compared by log-rank test. Cox regression model was applied in the univariate and multivariable analysis.

The results of Cox regression analysis in the DLBCL patients were combined to construct the nomograms for predicting 1-, 5-, and 10-year OS and DSS, respectively. The nomogram performance was assessed using Harrell's concordance index (C-index), which could estimate the discrimination between the predicted and actual survival. We also built the calibration curves to identify whether the predicted and actual survival were in agreement.

All statistical analysis was performed using R software. The R package included survival, survminer, rms, rmda, and ggplot2. Statistical significance was set at a two-sided *P*-value < 0.05.

## Results

### Demographics and Incidence of PHL Patients

**Figure 1:**
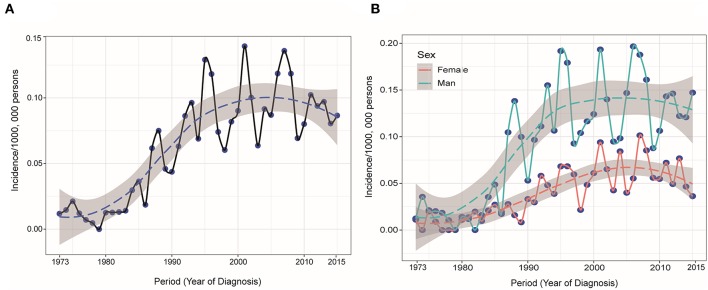
**(A)** Annual age-adjusted incidence of primary hepatic lymphoma was increasing from 1973 to 2015 and **(B)** this trend was more remarkable among male population.

The study identified 1,182 PHL patients from 1983 to 2015. The trend in incidence was relatively steady increasing from 1973 to 2015, with an APC of 2.6% (95% CI 2.0–3.2, *P* < 0.05) (Figure 1A). This trend was more remarkable among male population (Figure 1B). The annual age-adjusted incidence of PHL was 0.011/100 000 persons in 1973 and 0.015/100,000 persons in 1974. The incidence was 0.080 and 0.087/100,000 persons in 2014 and 2015, respectively. The mean age at diagnosis was 61.7 ± 17.1, with a wide range of 3–97 years. The whole cohort constituted of 732 (61.9%) males and 450 (38.1%) females. The majority of patients were White (82.0%) and unmarried (54.0%). The characteristics of these PHL patients are summarized in Table 1.

**Table 1:** Patient and tumor characteristics of primary hepatic lymphoma diagnosed in SEER 18 registries, 1983–2015.

**Characteristic**	**No. of patients**	**Percentage (%)**
**Total**	**1,182**	**100**
**Age at diagnosis, years**		
Mean ± SD	61.67 ± 17.12	
Median(rang)	63.0 (3.0–97.0)	
**Sex**		
Male	732	61.9
Female	450	38.1
**Race**		
White	969	82.0
Black	130	11.0
Others^a^	83	7.0
**Years of diagnosis**		
1983–1993	94	8.0
1994–2004	386	32.7
2005–2015	702	59.4
**Marital status**		
Married	544	46.0
Unmarried	638	54.0
**Classification**		
Hodgkin Lymphoma	27	2.3
Aggressive B cell NHL^b^	742	62.8
Indolent B cell NHL^c^	153	12.9
T cell NHL	42	3.6
NHL-NOS	118	10.0
Other/Unclassified	100	8.5
**Surgery**		
No	1,141	96.5
Performed	41	3.5
**Radiation**		
No	1,127	95.3
Performed	55	4.7
**Chemotherapy**		
No/unknown	405	34.3
Performed	777	65.7

*NHL, non–Hodgkin lymphoma; NOS, not otherwise specified*.

a *American Indian/Alaskan Native or Asian/Pacific Islander*.

b *Included diffuse large B cell lymphoma and Burkitt's lymphoma*.

c *Included follicular lymphoma, chronic lymphocytic leukemia/small lymphocytic lymphoma, lymphoplasmacytic lymphoma, and mucosal-associated lymphoid tissue*.

According to cell origin and clinical aggressiveness, there were 742 (62.8%) patients of aggressive B cell NHLs, 153 (12.9%) indolent B cell NHLs, 118 (10.0%) NHL–NOSs and 42 (3.6%) T cell NHLs. Histologically, the most prevalent subtype was diffuse large B cell lymphoma (DLBCL) (62.3 %) and NHL-NOS (10.9%), followed by mucosal-associated lymphoid tissue (MALT) (3.7%), Burkitt's lymphoma (3.4%), follicular lymphoma (2.6%), T cell lymphomas (2.5%), Hodgkin lymphoma (2.3%), chronic lymphocytic leukemia/small lymphocytic lymphoma (1.9%), and finally lymphoplasmacytic lymphoma (0.9%), anaplastic large cell lymphoma (0.9%). All of the PHL subtypes were found more prevalently among male patients than female, except for lymphoplasmacytic lymphoma (36.4%) and follicular lymphoma (45.2%). Although the median age for all patients was 63.0 years, patients with Burkitt's lymphoma had a much younger median age (34 years). The demographic and survival characteristics of all patients based on histological subtype was summarized in Table 2. Only a fraction received radiation therapy (4.7%) or surgery (3.5%). More than half of them received chemotherapy (65.7%). Chemotherapy-related deaths were not mentioned in the database.

**Table 2:** Patient characteristics according to the histological subtypes.

**Histology subtype**	***n* (%)**	**Median age, y, (range)**	**Male, *n* (%)**	**Survival**			
				**Median OS, m**	**5-year OS**	**Median DSS, m**	**5-year DSS**
All patients	1,182	63.0 (3–97)	732 (61.9)	13.0	39.6%	37.0	47.9%
DLBCL	736 (62.3)	64.0 (2–97)	471 (63.9)	12.0	38.6%	33.0	46.7%
LPL	11 (0.9)	60.0 (27–88)	4 (36.4)	12.0	NR	22.0	NR
ALCL	11 (0.9)	47.0 (23–80)	8 (72.7)	2.0	NR	2.0	NR
CLL/SLL	22 (1.9)	66.0 (25–93)	13 (59.1)	53.0	49.5%	58.0	54.5%
FL	31 (2.6)	67.0 (41–92)	14 (45.2)	65.0	58.2%	34.0	69.6%
BL	40 (3.4)	34.0 (5–87)	32 (80.0)	52.0	51.8%	69.0	57.3%
MALT	44 (3.7)	66.0 (44–88)	24 (54.5)	109.0	57.3%	153.0	67.2%
T cell lymphoma	30 (2.5)	59.0 (21–92)	20 (66.7)	8.0	34.7%	11.0	69.1%
NHL-NOS	129 (10.9)	62.0 (13–93)	75 (58.1)	8.0	33.4%	11.0	41.1%
HL	27 (2.3)	66.0 (31–90)	14 (51.9)	71.*o*	51.5%	85.0	60.1%
Others	101	64.0 (11–94)	57 (56.4)	5.0	32.5%	7.0	38.9%

*n, number of cases; y, year; m, month; OS, overall survival; DSS, disease-specific survival; NHL, non–Hodgkin lymphoma; NOS, not otherwise specified; NR, not reached; DLBCL, diffuse large B cell lymphoma; FL, Follicular lymphoma, BL, Burkitt's lymphoma; CLL/SLL, chronic lymphocytic leukemia/small lymphocytic lymphoma; LPL, Lymphoplasmacytic lymphoma; ALCL, Anaplastic large cell lymphoma; MALT, Mucosal-associated lymphoid tissue*.

### Survival Analysis

**Figure 2:**
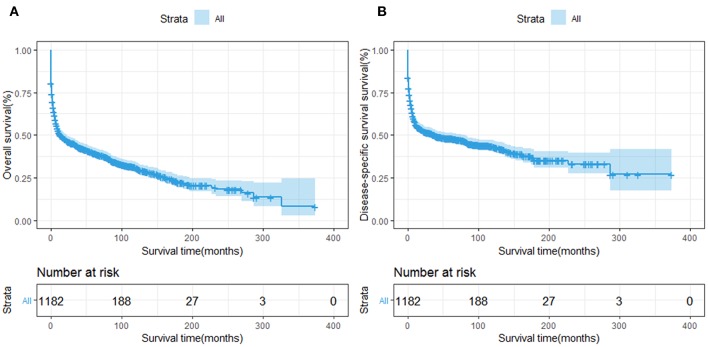
Survival analysis of primary hepatic lymphoma: **(A)** OS and **(B)** DSS were shown for all patients.

**Figure 3:**
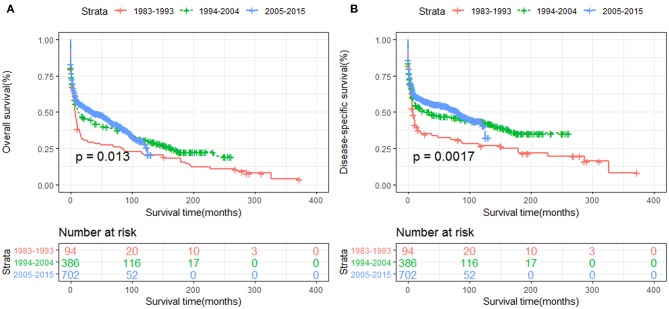
The survival of primary hepatic lymphoma improved significantly over the past four decades: **(A)** OS and **(B)** DSS.

The OS and DSS of all PHL patients were illustrated in Figures 2A,B. A total of 770 patients died by the end of follow-up, 606 deaths were disease-specific, attributable to PHL. Kaplan-Meier survival analysis indicated that the median OS and DSS for all was 13.0, 37.0 months, respectively. The 1-, 5-, and 10-year OS were 50.85, 39.6, and 30.4%, respectively. The 1-, 5-, and 10-year DSS were 55.3, 47.9, and 43.3%, respectively. The survival improved significantly over the past four decades, OS and DSS for patients diagnosed in 1994–2004 and for patients diagnosed in 2005–2015 was both improved significantly compared to that of patients diagnosed in 1983–1993 (both *P* < 0.001) (Figures 3A,B).

**Figure 4:**
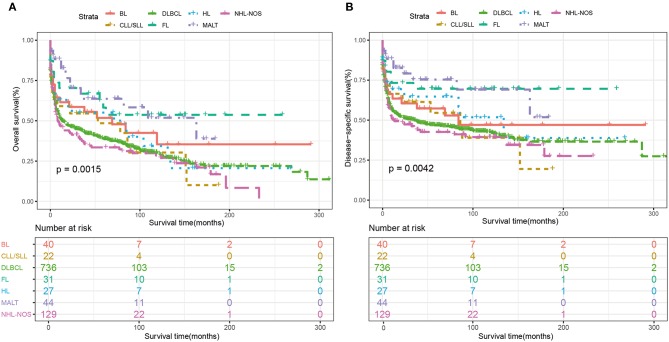
Kaplan-Meier survival analysis of overall survival according to the main histological subtypes. **(A)** OS and **(B)** DSS. BL, Burkitt's lymphoma; CLL/SLL, chronic lymphocytic leukemia/small lymphocytic lymphoma; DLBCL, diffuse large B cell lymphoma; FL, Follicular lymphoma; HL, Hodgkin lymphoma; MALT, Mucosal-associated lymphoid tissue; HL, non-Hodgkin lymphoma; NOS, not otherwise specified.

Among the whole cohort, the best 5-year OS and DSS rates were observed among follicular lymphoma (OS: 58.2%, DSS: 69.6%), MALT (OS: 57.3%, DSS: 67.2%) and Burkitt's lymphoma (OS:51.8%, DSS:57.3%). The 5-year OS rates of DLBCL was 38.6%, which was similar to NHL-NOS (33.4%) and T cell NHL (34.7%) (Table 2). Additionally, the Kaplan-Meier curves of OS and DSS for the main subtypes of PHL were shown in Figure 4.

**Figure 5:**
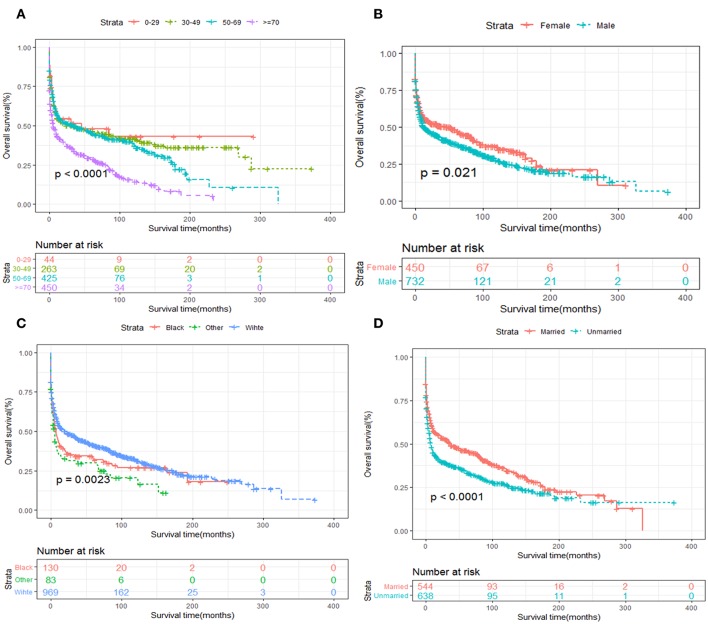
OS analysis of primary hepatic lymphoma stratified by **(A)** age, **(B)** sex, **(C)** race, **(D)** marital status.

**Figure 6:**
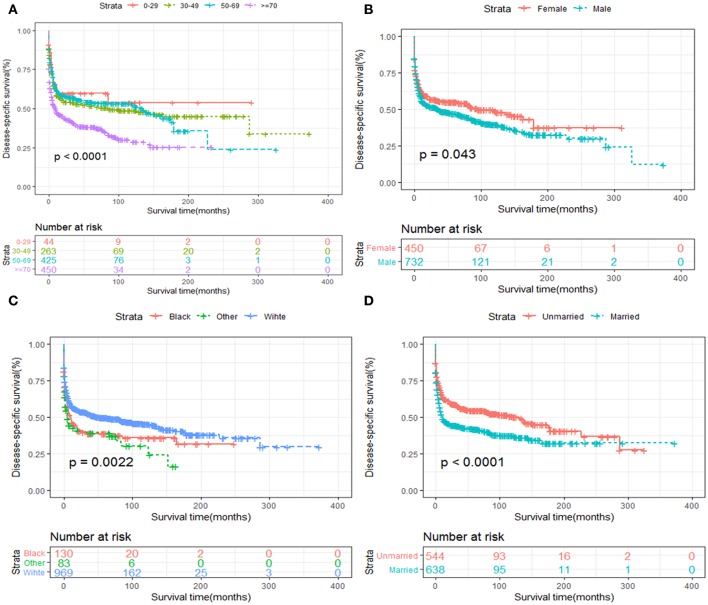
DSS analysis of primary hepatic lymphoma stratified by **(A)** age, **(B)** sex, **(C)** race, **(D)** marital status.

Kaplan-Meier survival analysis of patients stratified by age, sex, race, years of diagnosis, marital status and treatment strategies were also performed. We revealed that elder age was significantly associated with inferior OS and DSS (Figures 5A, 6A). Women tended to enjoy longer OS and DSS than men (Figures 5B, 6B). Univariate analysis also demonstrated a better prognosis for patients who were White (Figures 5C, 6C) and married (Figures 5D, 6D).

**Figure 7:**
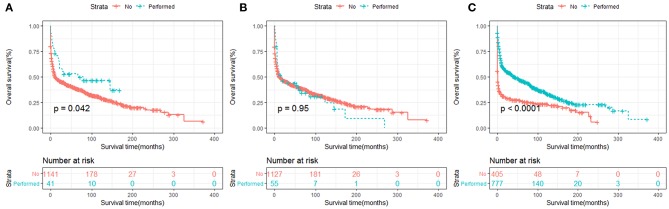
OS analysis of primary hepatic lymphoma stratified by treatment strategies: **(A)** surgery, **(B)** radiation, **(C)** chemotherapy.

**Figure 8:**
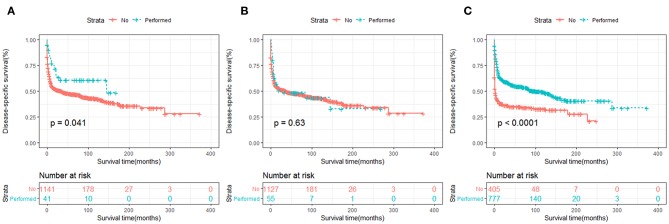
DSS analysis of primary hepatic lymphoma stratified by treatment strategies: **(A)** surgery, **(B)** radiation, **(C)** chemotherapy.

In terms of treatment strategies, patients who received surgery (Figures 7A, 8A) or chemotherapy (Figures 7C, 8C) had significantly better OS and DSS than those who did not. However, radiation therapy did not significantly influence OS or DSS (Figures 7B, 8B).

Multivariable Cox regression analysis was performed to identify the independent prognostic factors for OS and DSS. The results indicated that age, sex, race, marital status, histological subtype, surgery, and chemotherapy were independently able to predict both OS and DSS (Table 3).

**Table 3:** Multivariable Cox regression analysis of the independent prognostic factors for OS and DSS among primary hepatic lymphoma patients.

**Variables**	**Overall survival**			**Disease-specific survival**		
	**HR**	**95% CI**	***P***	**HR**	**95% CI**	***P***
**Age at diagnosis, years**						
0–29	Reference			Reference		
30–49	1.24	0.77–1.97	0.371	1.42	0.84–2.41	0.187
50–69	1.78	1.12–2.81	0.014	1.81	1.07–3.05	0.025
≥70	2.96	1.88–4.68	<0.001	3.04	1.81–5.09	<0.001
**Sex**						
Female	Reference			Reference		
Male	1.46	1.24–1.71	<0.001	1.44	1.19–1.72	<0.001
**Race**						
Black	Reference			Reference		
White	0.76	0.60–0.96	0.019	0.75	0.58–0.96	0.025
Others^a^	1.08	0.77–1.51	0.642	1.11	0.77–1.59	0.589
**Years of diagnosis**						
1983–1993	Reference			Reference		
1994–2004	0.67	0.52–0.87	0.002	0.59	0.45–0.79	<0.001
2005–2015	0.56	0.43–0.73	<0.001	0.48	0.36–0.64	<0.001
**Marital status**						
Married	Reference			Reference		
Unmarried	1.41	1.21–1.64	<0.001	1.49	1.26–1.78	<0.001
**Classification**						
Hodgkin Lymphoma	Reference			Reference		
Aggressive B cell NHL	1.12	0.69–1.80	0.634	1.28	0.72–2.28	0.397
Indolent B cell NHL	0.78	0.47–1.31	0.359	0.88	0.47–1.64	0.709
NHL-NOS	0.93	0.55–1.56	0.782	1.06	0.57–1.99	0.836
T cell NHL	2.22	1.22–4.03	0.009	2.75	1.38–5.51	0.004
Other/unclassified	0.98	0.58–1.66	0.933	1.04	0.55–1.97	0.895
**Surgery**						
No/unknown	Reference			Reference		
Performed	0.74	0.58–0.94	0.02	0.60	0.42–0.86	0.008
**Radiation**						
No/unknown	Reference			Reference		
Performed	1.15	0.83–1.59	0.406	1.05	0.72–1.54	0.774
**Chemotherapy**						
No/unknown	Reference			Reference		
Performed	0.45	0.39–0.53	<0.001	0.43	0.36–0.51	<0.001

a *American Indian/Alaskan Native or Asian/Pacific Islander*.

### Construction and Validation of the Nomograms

Giving that the main histology subtype of PHL was DLBCL, hence we focused on these patients and aimed to develop a new prediction model specifically for patients with DLBCL. First of all, we performed the univariate and multivariate Cox regression analysis to identify the independent prognostic factors for OS and DSS, respectively. The results of the univariate and multivariate analysis were listed in Table 4. Univariate analyses demonstrated that age at age, marital status, surgery, and chemotherapy were associated with OS. Regarding DSS, surgery lost its significance, while other factors continued to be significant. These significant factors derived in the univariate Cox regression analysis were then included in the multivariable analysis. And multivariate analysis demonstrated that age, marital status and chemotherapy were independent prognostic factors for OS. Regarding DSS, age was excluded, while marital status and chemotherapy remained significant indicators.

**Table 4:** Univariate and multivariate Cox regression analysis of each factor's ability in predicting OS and DSS among DLBCL patients.

	**Overall Survival**		**Disease-Specific Survival**	
	**HR (95% CI)**	***P***	**HR (95% CI)**	***P***
**(A) UNIVARIATE ANALYSES**				
**Age at diagnosis**				
0–29 vs. 30–49	1.00 (0.50–1.99)	0.999	1.08 (0.52–2.23)	0.838
0–29 vs. 50–69	1.36 (0.70–2.67)	0.365	1.20 (0.58–2.45)	0.625
0–29 vs. ≥ 70	2.12 (1.09–4.13)	0.026	1.84 (0.91–3.75)	0.090
**Sex**				
Female vs. Male	0.93 (0.77–1.12)	0.433	0.93 (0.75–1.14)	0.468
**Race**				
Black vs. White	1.25 (0.83–1.89)	0.292	1.08 (0.69–1.71)	0.726
Black vs. Others^a^	0.86 (0.65–1.14)	0.283	0.76 (0.56–1.03)	0.072
**Marital status**				
Married vs. Unmarried	1.26 (1.05–1.51)	0.0134	1.43 (1.17–1.76)	<0.001
**Surgery**				
No/unknown vs. Performed	0.57 (0.33–0.99)	0.0474	0.57 (0.30–1.06)	0.075
**Radiation**				
No/unknown vs. Performed	0.99 (0.67–1.46)	0.969	0.91 (0.58–1.42)	0.665
**Chemotherapy**				
No/unknown vs. Performed	0.34 (0.28–0.41)	<0.001	0.34 (0.27–0.42)	<0.001
**(B) MULTIVARIATE ANALYSES**				
**Age at diagnosis**			/	
0–29 vs. 30–49	1.21 (0.61–2.41)	0.586	/	/
0–29 vs. 50–69	1.83 (0.93–3.62)	0.080	/	/
0–29 vs. ≥ 70	2.53 (1.29–4.96)	0.006	/	/
**Marital status**				
Married vs. Unmarried	1.34 (1.11–1.62)	0.002	1.33 (1.09–1.64)	0.005
**Surgery**			/	
No/unknown vs. Performed	0.63(0.36–1.09)	0.100	/	/
**Chemotherapy**				
No/unknown vs. Performed	0.36 (0.30–0.44)	<0.001	0.35 (0.28–0.43)	<0.001

a *American Indian/Alaskan Native or Asian/Pacific Islander*.

**Figure 9:**
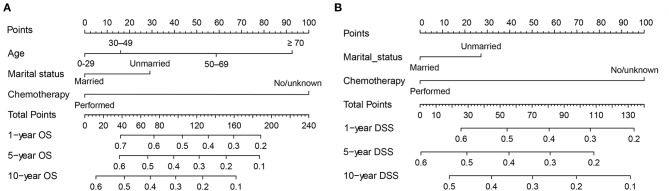
Nomograms for predicting the 1-, 5-, and 10-year OS **(A)** and DSS **(B)** of primary hepatic lymphoma patients.

**Figure 10:**
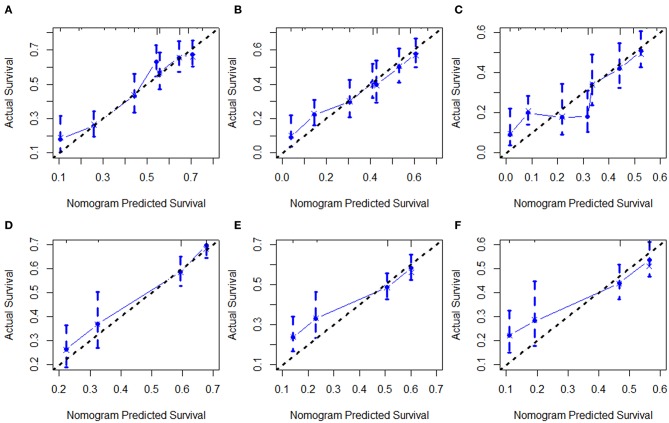
Calibration curves of the nomogram for 1-,5-, and 10-year OS **(A–C)** and DSS **(D–F)**.

Next, all independent prognostic factors of the Cox regression analysis were integrated to construct the prognostic nomogram. Figure 9A showed the OS nomogram at the 1-, 5-, and 10- year, and Figure 9B presented the DSS nomogram at 1-, 5-, and 10- year. By adding up the scores related to each parameter and projecting the overall scores to the bottom scale, the possibility of OS and DSS at 1-, 5-, and 10- year could be estimated. Furthermore, we used the C-index and the calibration curves to evaluate the performance of the established nomograms. The C-index for nomogram predictions of OS and DSS were 0.689 (95% CI 0.661–0.716) and 0.667 (95% CI 0.638–0.696), respectively, indicating that the newly established nomograms were considerably accurate. Analogously, the calibration curves in the training and validation cohorts showed excellent consistency between the nomogram prediction and actual OS and DSS at 1-, 5-, and 10- year (Figure 10).

## Discussion

Because of its rarity, few data on the incidence, characteristics and survival of PHL are available. Therefore, the current study conducted an analysis of a population-based cohort of PHL patients from SEER registries across the United States. Several important conclusions can be derived according to our study. We observed an upward trend in PHL incidence throughout the past four decades, with an APC of 2.6% (95% CI 2.0–3.2, *P* < 0.05), which might be partly due to an increasing awareness of the disease as a unique entity over time. Likewise, increasing trends of OS and DSS were observed over time, which might benefit from the current comprehensive treatment strategies. In particular, the rapidly developing molecule-targeted treatment for CD20-positive B lymphoma has further improved prognosis in recent years (6–8).

The etiology of PHL remains largely unknown, although viral infections such as HBV, HCV, Epstein-Barr virus, human T-lymphotropic virus, liver cirrhosis, primary biliary cirrhosis, immunosuppressive therapy, as well as autoimmune disease have been implicated (9–12). Among all these, Hepatitis C is observed in 40–60% of patients with PHL (13). However, potential roles of HCV in lymphomagenesis remain hypothetical. The HCV genome cannot integrate into the host cell genome thus cannot make the cell transformation, indicating that the malignant transformation probably occurs in indirect manners. A persistent HCV infection leads to a chronic stimulation of B-cells, producing a polyclonal expansion of these cells; the occurrence of additional genetic alterations may trigger a cell subset with autonomous growth, leading to their progressive proliferation and accumulation (14–17). In addition, insufficient evidence exists on the association between HBV infection and PHL pathogenesis. Aozasa et al. (18) have suggested that chronic antigenic stimulation induce by persistent HBV infection might continue critically in the PHL pathogenesis. Although it remains uncertain to what extent HBV contributes to the pathogenesis and progression of PHL, an altered immune system within the host environment might play a vital role that cannot be ignored (10, 19).

The present study represented the largest and latest analysis of PHL to date. We revealed that B cell NHL including aggressive B cell NHL, indolent B NHL accounted for ~76% of PHL, and the most common histopathological diagnosis was DLBCL, which was observed in 736 (62.3%) patients. This result accorded with the prior Western (20, 21) and Asian reports (22). In a retrospective review of 59 PHL patients, more than half were B cell lineage (33 patients, 62%) and 16 were T cell (30%) (23). In our study, the proportion of T-cell NHL was relatively low (3.6%) compared with previous reports (5–10%) (24). According to literature, the most observed T-cell NHL in the liver were mature T-cell lymphoma-NOS and peripheral T-cell lymphoma (25), followed by anaplastic T-cell lymphoma (26), and hepatosplenic T-cell lymphoma (27). The incidence of aggressive T-cell NHLs rose obviously among Asian (8.7%) and Black (6.0%) population, in comparison to the White (2.8%). This phenomenon would, to some extent, mirror increased exposure to etiological factors, such as hepatitis C Virus (HCV) and Epstein-Barr virus (EBV) in Asian and African countries.

The mean age at diagnosis in this study was 61.67 ± 17.12 years, which is slightly older than previous reports that PHL occurs typically in the fifth decade (28). Our results showed advanced age was associated with worse prognosis for both OS and DSS. The male to female ratio was 1.6:1 among PHL patients, consisting with previous reports that men are more vulnerable than women. It indicates a potential role for sex hormones working in the pathogenesis of PHL and Recently, multiple studies have demonstrated that estrogen served a protective role through decreasing the level of serum IL-6 interleukin-6 and thereby inhibited lymphoma in female patients (29–31). This phenomenon may in part explain the relatively low incidence of PHL in female. Interestingly, our study found that female sex was a favorable prognostic factor for OS and DSS. Female patients have been demonstrated better response to chemotherapy than the male (32). This may partly contribute to their survival advantages. In addition, the prognosis of PHL varied significantly by race and marital status, with white or married experiencing longer survival.

At present, there are no common and standard protocols or guidelines for the treatment of PHL. Surgery, chemotherapy, or radiotherapy alone, or in combination had been commonly used. The treatment strategy for PHL is determined by several factors, such as patient's age, stage of disease, histologic subtypes and clinical signs and symptoms at diagnosis. Actually, chemotherapy is widely used in patients with high risk of surgery and do not qualify surgical treatment, such as cases with unresectable tumors, or diffuse hepatic infiltration, cases with advanced disease involving extrahepatic tissues, and cases with highly malignant histological subtypes, etc. Indeed, for these cases, the average survival time is also relatively poor (6.0 months) (3). The conventional treatment for non-Hodgkin lymphomas is anthracycline based regimen (CHOP protocol) and since the 2000s, rituximab, an anti-CD20 monoclonal antibody, has been added to this regimen with an improved complete remission and tolerable adverse events. Remarkably, HCV infection does not seem to affect the chemotherapy response and the tolerance to chemotherapy (33). In addition, on the basis of chemotherapy, conducting radiotherapy can achieve longer survival time than chemotherapy alone.

However, the role of surgical treatment is not fully clarified. Generally, surgical resection is indicated if the patient is in good condition with a less severe disease. Several reports have demonstrated that surgical resection alone or followed by chemotherapy or radiotherapy can provide the good clinical remission. And the overall post-operative survival time reached 10 years (34–36). This suggested that first line surgical resection combined with chemotherapy or radiotherapy might produce a satisfactory curative effect for PHL and need to go further in future researches. However, the treatment decision-making for PHL warrants further investigation, as most analysis is based on retrospective studies with small sample size and a randomized controlled trial is still lacking.

The nomogram has currently been proposed as an important prediction model in clinical management (37). In this study, age, marital status, and chemotherapy were found to be independent prognostic factors for OS and DSS for the DLBCL cases, and nomograms based on these factors were built to predict 1-, 5-, and 10-year OS and DSS. Validation of the nomogram was necessary to reduce over-fitting model and determining general applicability (38, 39). In our study, discrimination was demonstrated by the obviously higher C-index of the nomogram. Calibration curves exhibited satisfactory agreement between the predicted and the actual survival in the entire cohort. Using these nomograms, we could easily and accurately predict individual survival probability at certain time and make reasonable follow-up schedules. However, the current nomograms were constructed and validated in the same database, therefore prospective validation of the nomograms in another independent dataset is warranted for reliable evaluation.

There are several limitations inherent to this study. First, our study was retrospective, and subject to unavoidable biases. Secondly, there are many other variables that could impact survival, such as international prognostic index (IPI), B symptoms, commodities, and several bio-markers (40). However, the SEER database did not record data regarding these variables, and thus those potential prognostic variables were not integrated into our nomograms. Thirdly, although the diagnosis for PHL was restricted in our study, the lymphoid neoplasms primarily limited to liver diagnosed by pathology, with no history of a prior or concurrent tumor diagnosis. Actually, information from the SEER database could not satisfy the strict diagnostic criteria for PHL proposed by Caccamo et al. (41). And some patients were not actually PHL and they might be cases of extrahepatic diseases with secondary liver involvement. However, there is not a uniform and accepted diagnose criterion of this disease currently, and some have considered cases as being primary, describing preponderant liver localization, even in the presence of extrahepatic diseases (24, 26). Finally, the interval of time of PHL patients enrolled in the study was quite long (32 years), and within this interval many classification, staging systems have changed (with introduction of novel imaging standards) as well as detection strategies. Therefore, this might have influenced the incidence as well as the prognosis and treatment, and we partially addressed this issue by dividing the time of diagnosis into1983–1993, 1994–2004, and 2005–2015. Given above, the results of our analysis might be interpreted with caution. Nevertheless, based on a large population, the SEER database remains a valuable source in studying such rare lymphoma despite these limitations. Our analysis still provided important insights for PHL, and useful information on incidence, prognostic factors and survival among PHL patients.

In conclusion, the current population-based study showed that PHL are a rare type of lymphoma with increasing incidence trend, particularly among male population. Surgery and chemotherapy were associated with better survival and should be recommend for PHL patient. We also constructed two robust and accurate nomograms, which might assist clinicians to estimate prognosis accurately and establish individualized tracking programs.

## Data Availability Statement

Publicly available datasets were analyzed in this study. This data can be found in the SEER database (https://seer.cancer.gov/).

## Author Contributions

S-LZ, Z-MW, and L-SW contributed to the conception, design, and drafted the manuscript. S-LZ, XW, and ZG analyzed the data. S-LZ, CC, Q-WR, and L-SW contributed with a critical revision of the manuscript.

## Conflict of Interest

The authors declare that the research was conducted in the absence of any commercial or financial relationships that could be construed as a potential conflict of interest.
